# miRNAs in Tuberculosis: New Avenues for Diagnosis and Host-Directed Therapy

**DOI:** 10.3389/fmicb.2018.00602

**Published:** 2018-03-29

**Authors:** Naveed Sabir, Tariq Hussain, Syed Zahid Ali Shah, Antonio Peramo, Deming Zhao, Xiangmei Zhou

**Affiliations:** State Key Laboratories for Agrobiotechnology, Key Laboratory of Animal Epidemiology of the Ministry of Agriculture, National Animal Transmissible Spongiform Encephalopathy Laboratory, College of Veterinary Medicine, China Agricultural University, Beijing, China

**Keywords:** *Mycobacterium tuberculosis*, miRNA expression, immune regulation, autophagy, apoptosis, biomarker, nanoparticles, host directed therapy

## Abstract

Tuberculosis (TB) is one of the most fatal infectious diseases and a leading cause of mortality, with 95% of these deaths occurring in developing countries. The causative agent, *Mycobacterium tuberculosis* (*Mtb*), has a well-established ability to circumvent the host’s immune system for its intracellular survival. microRNAs (miRNAs) are small, non-coding RNAs having an important function at the post-transcriptional level and are involved in shaping immunity by regulating the repertoire of genes expressed in immune cells. It has been established in recent studies that the innate immune response against TB is significantly regulated by miRNAs. Moreover, differential expression of miRNA in *Mtb* infection can reflect the disease progression and may help distinguish between active and latent TB infection (LTBI). These findings encouraged the application of miRNAs as potential biomarkers. Similarly, active participation of miRNAs in modulation of autophagy and apoptosis responses against *Mtb* opens an exciting avenue for the exploitation of miRNAs as host directed therapy (HDT) against TB. Nanoparticles mediated delivery of miRNAs to treat various diseases has been reported and this technology has a great potential to be used in TB. In reality, this exploitation of miRNAs as biomarkers and in HDT is still in its infancy stage, and more studies using animal models mimicking human TB are advocated to assess the role of miRNAs as biomarkers and therapeutic targets. In this review, we attempt to summarize the recent advancements in the role of miRNAs in TB as immune modulator, miRNAs’ capability to distinguish between active and latent TB and, finally, usage of miRNAs as therapeutic targets against TB.

## Introduction

Tuberculosis (TB) caused by *Mycobacterium tuberculosis (Mtb)* is one of the most fatal infectious diseases (Dye and Williams, 2010). Current estimations show that 1/3 of the world population is latently infected with *Mtb*. However, only 5–10% of infected people develop active TB in their lifetime (Bhatt and Salgame, 2007). It is the leading infectious cause of deaths, exceeding HIV/AID, resulting in approximately 1.3 million human deaths in 2016 (WHO, 2017). *Mtb* is an intracellular pathogen and its ability to survive inside host macrophages and tubercle granulomas constitutes the major attribute of its virulence. Macrophages are an important component of the host innate immune response and are able to generate a variety of antimicrobial molecular responses, such as antibacterial peptides, hydrolases and toxic reactive oxygen and nitrogen intermediates (Fenton and Vermeulen, 1996). Research on how *Mtb* is capable of surviving in this antibacterial environment remains an area of interest for many research groups. Accumulating evidence suggests that *Mtb* is capable of modulating cellular processes such as cytokine production, autophagy, apoptosis, MHC class II expression and phagolysosome maturation in macrophages and dendritic cells (Ahmad, 2011). Currently, many studies have reported that most of the above mentioned cellular mediated immune responses of eukaryotic cells are under the control of miRNAs (Bartel, 2004; Giraldez et al., 2005) and it is now established that modulation of miRNAs expression associated with these biological processes is one of the important strategies implemented by bacterial pathogens to survive inside host immune cells (Das et al., 2016).

miRNAs are small, non-coding RNAs having an important function at the post-transcriptional level to regulate gene expression (O’Connell et al., 2010; Watanabe and Kanai, 2011). Binding of miRNAs to their complementary sequences in the 3′ untranslated region (3′ UTR) of their respective protein coding mRNA targets results in transcript degradation or translational inhibition (Maute et al., 2014). The human genome may encode more than two thousands potentially functional miRNAs (Kozomara and Griffiths-Jones, 2014; Hammond, 2015) and it is estimated that one-half of all protein-coding transcripts are subjected to miRNA regulation (Bartel, 2009; Krol et al., 2010; Watanabe and Kanai, 2011; Vera et al., 2013). Each miRNA may suppress multiple genes and one mRNA can be targeted by multiple miRNAs. Therefore, disease-associated miRNAs represent a new class of diagnostic markers or therapeutic targets (Mayr et al., 2007; Pillai et al., 2007; Subramanian and Steer, 2010; Jackson and Levin, 2012; Mendell and Olson, 2012; Nana-Sinkam and Croce, 2013). Moreover, some bioinformatics studies hint at the existence of tens of thousands of short non-coding RNAs similar to miRNAs with the potential to regulate expression of most or all human genes, although it is unclear whether all of the small RNAs are functional or they are just fragments of larger RNAs (Brameier and Wiuf, 2007; Dieci et al., 2007; Bentwich, 2008). Previously, it was believed that transcription factors (TFs) are responsible for the regulation of gene expression for various cell phenotypes and responses of cells to environmental stimuli. But with the discovery of miRNAs, it is speculated that miRNAs, in combination with TFs, control the expression of thousands of mammalian genes, a process described as ‘tuning’ the transcriptional network (Zhou et al., 2007; Fabian et al., 2010). Some miRNAs, like miR-16, are widely expressed in body tissues while others are tissue or developmental stage specific (Poy et al., 2004). The tissue-specific expression of miRNAs is suggestive of their role in cell differentiation and function (Shi et al., 2010; Pauli et al., 2011).

It has been established that both adaptive and innate immune responses are regulated by miRNAs. For example, in the adaptive immune response, the differentiation of B cells, antibody generation, and T cell development and function are controlled by miRNAs (Singh et al., 2013). A number of recent studies described the regulation of mammalian miRNAs in response to bacterial infection (De Flora and Bonanni, 2011). Innate immune cell activation requires miRNAs, including miR-155, miR-146a, miR-21, and miR-9 (Belver et al., 2011). Components of inflammatory and immune pathways are regulated by miRNAs under the challenge of mycobacterial infections (Liu et al., 2011; Chatterjee et al., 2011). For example, TNF biosynthesis is inhibited by miR-125b in human alveolar macrophages during *Mtb* infection (Rajaram et al., 2011). Furthermore, many research groups have reported the differential expression of miRNAs in host cells challenged with *Mtb*, indicating the important role of these miRNAs in regulating the immune response in TB and proposing the miRNAs as potential biomarkers (Wang et al., 2011; Qi et al., 2012; Wu et al., 2012; Zhang et al., 2013). Many of these studies also provide an insight into the role of these differentially expressed miRNAs in TB, but a detailed understanding of the role of these miRNAs in the anti-mycobacterial response is a matter of highest interest for future investigations. These studies also provide a good foundation for the development of reliable biomarkers and therapeutic targets for TB.

Autophagy plays a pivotal role in controlling the bacterial load during TB (Deretic et al., 2013; Richetta and Faure, 2013). However, *Mtb* has evolved strategies to survive in macrophages by evading delivery to the lysosomes (Hmama et al., 2015) and like other intracellular pathogens *Mtb* also has the ability to exploit host degradative processes to breakdown cellular macromolecules into simple nutrients for its survival and multiplication (Steele et al., 2015). Similarly, cell apoptosis is also one of the important host defense mechanisms through which macrophages control *Mtb* infection (Weiss and Schaible, 2015). *Mtb* is able to inhibit phagolysosome biogenesis, inhibition of apoptosis as well as autophagy due to presence of lipoarabinomannan in its cell wall which is a major immunomodulatory lipoglycan (Vergne et al., 2015). The emerging roles of miRNAs in regulating *Mtb*–induced autophagy and apoptosis have attracted increased attention in recent years. Appreciating the potential to be exploited in host-directed therapies designed to control autophagy and apoptosis, many groups have focused their research on miRNAs (Ouimet et al., 2016; Zhang et al., 2016; Guo et al., 2017). These recent studies have opened a new avenue to exploit miRNAs in HDT. In the current review, we will summarize recent advances in the understanding of differential expression of miRNAs and their role in TB, and their potential to be used as biomarkers and therapeutic targets for diagnosis and treatment of this deadly disease.

## Differential Expression of miRNAs in Different Cell Types Following Mtb Infection

Several studies have addressed the differential expression of miRNAs as reflecting disease prediction and progression *in vivo* and *in vitro*. For example, differentially expressed miRNAs have a close relationship with disease progression to hepatocellular carcinoma in both hepatitis B and C (Ura et al., 2009). Recently, attempts have been made to determine the effects of *Mtb* infection on the expression of miRNAs in the host (Ghorpade et al., 2012; Vegh et al., 2015; Zheng L. et al., 2015). In a recent study, mouse bone marrow derived macrophages (BMDMs) infected with *Mtb* showed up-regulation of miR-155 (Kumar et al., 2015) while infection of peripheral blood mononuclear cells (PBMCs) derived macrophages with the same pathogen led to down-regulation of miR-155 expression (Rajaram et al., 2011). This means different cell types may respond differently upon infection with *Mtb*. Sharbati et al. (2011) reported overexpression of let-7e, miR-29a and miR-886-5p in human monocyte derived macrophages (MDMs) in response to mycobacterial infection. Integrated analysis of microRNA and mRNA expression as well as target prediction revealed caspases 3 and 7 as potential targets of let-7e, and miR-29a, respectively. Liu et al. (2012) reported that differentially expressed hsa-mir-21 inhibits expression of two genes encoding vitamin D–dependent antimicrobial peptides, CAMP and DEFB4A, in human monocytes derived from leprosy patients. This inhibition is carried out by direct downregulation of Toll-like receptor (TLR) 2/1 heterodimer (TLR2/1)-induced CYP27B1 and IL1B expression as well as indirect upregulation of IL-10. Furci et al. (2013) studied *Mtb*-induced miRNA expression profile in primary human macrophages infected with virulent *Mtb* H37Rv and avirulent *M. bovis* BCG and showed that macrophages differentially expressed miRNAs, including miR-155, miR-146a, miR-145, miR-222^∗^, miR-27a, and miR-27b. In this study, miR-222^∗^, miR-27a, and miR-27b, which have been reported to control inflammatory response and lipid metabolism (McGregor and Choi, 2011; Graff et al., 2012) were significantly downregulated. miR-145, which has been reported to induce apoptosis (Spizzo et al., 2010), was also downregulated, in line with a reduced capacity of virulent *Mtb* strain to induce apoptosis (Keane et al., 2000; Arcila et al., 2007; Starczynowski et al., 2010; O’Neill et al., 2011). Downregulation of miR-145 results in overexpression of its targets and inhibition of apoptosis (Starczynowski et al., 2010).

Das et al. (2013) analyzed global changes in the miRNA expression profile using microarrays and found that nine miRNA genes (miR-30a, miR-30e, miR-155, miR-1275, miR-3665, miR-3178, miR-4484, miR-4668-5p, and miR-4497) were differentially expressed in THP-1 cells infected with *Mtb* H37Rv or *Mtb* H37Ra strains. These differentially expressed miRNAs perform various important functions. miR-30e is activated by β-catenin (Schepeler et al., 2012), while miR-30a inhibits the epithelial to mesenchymal transition (Kumarswamy et al., 2012). miR-1275 is associated with liver metastases of cancer (Kahlert et al., 2011) and miR-155 binds a negative regulator associated with TNF-α production (Rajaram et al., 2011). Spinelli et al. (2013) examined PBMCs and pleural fluid mononuclear cells (PFMCs), and reported that miRNAs expression is associated with IL-6 levels, a cytokine playing a substantial role in TB immunopathology. Lin et al. (2015) showed that the Beijing/W TB strains repressed a number of miRNAs in human macrophages as compared to the non-Beijing/W TB strains, which might reflect their virulence characteristics in altering the host response. Two other research groups identified a series of miRNA differentially expressed in PBMCs of Chinese patients with pulmonary TB by using miRNA expression profiling. Liu et al. (2011) showed that the expression of several miRNAs was significantly altered in patients with active TB, with miR-144^∗^ being mainly expressed in T cells. Functional analysis showed that miR-144^∗^ inhibits the secretion of two important cytokines, INF-γ and TNF-α, and also reduces T cell proliferation. Wang et al. (2011) demonstrated that miR-424 and miR-365 levels were significantly raised in patients with active TB compared to healthy controls. In recent years, many other studies have reported the differential expression of miRNAs in different cell types in response to mycobacterial infection (Ni et al., 2014; Zhang et al., 2015). The detailed role of these differentially expressed miRNAs in anti-mycobacterial response is a matter of high interest for many research groups. Furthermore, these studies also provide a good foundation for the development of reliable biomarkers for TB diagnosis.

## miRNAs as Potential TB Biomarkers

An essential method to effectively control the spread of TB is to diagnose it at an early stage. Currently used test systems are insufficient and unable in practice to discriminate between active TB and latent TB infection (LTBI). Altered miRNA expression profiles may help differentiate between active TB and LTBI and can also act as reliable biomarkers for the diagnosis of the disease. This potential of miRNAs has received much attention in recent years, with several *in vivo* and *in vitro* studies proposing miRNAs as potential biomarkers (Miotto et al., 2013; Weiner et al., 2013) although suitable miRNA biomarkers have not been established yet (Walz et al., 2011) (**Table 1**). In a study using human PBMCs, the authors suggested that differentially expressed miRNAs combined with predicted differentially expressed mRNAs from the same whole genome transcriptional profiling may be used as a new way to differentiate among active TB, LTBI and healthy controls (Xu et al., 2013). Maertzdorf et al. (2012) identified a cluster of differentially expressed miRNAs in patients having active TB and sarcoidosis. Interestingly, strong correlations between miRNAs and gene expression were found. Similarly, lipomannan from virulent *Mtb* stimulated the expression of miR-125b in human macrophages while lipomannan from avirulent *M. smegmatis* -resulted in increased expression of miR-155 (Rajaram et al., 2011). These reports revealed that components from related bacterial species, with different virulence, lead to differential expression of miRNAs and consequently modulated the immune response.

**Table 1:** Differential expression of miRNAs in tuberculosis and their potential as biomarkers.

Species examined	Type of tissue/cells examined	Candidate biomarkers	Reference
Human	PBMCs	has-miR-21^∗^ and has-miR-26b	Xu et al., 2013
Human	Macrophages	miR-125b and miR-155	Rajaram et al., 2011
Human	Macrophages	miR-29a and miR-361-5p	Draz et al., 2014
Human	Macrophages	miR-31	Wang et al., 2015
Human	Serum	miR-4433b-5p, miR-424-5p, and miR-199b-5p	Wang et al., 2016
Human	Whole blood	hsa-miR-21 hsa-miR-7f-1^∗^	Latorre et al., 2015
Human	Serum	miR-361-5p, miR-889, and miR-576-3p	Qi et al., 2012
Human	Whole blood	miR-1, miR-155, miR-31, miR-146a, miR-10a, miR-125b, miR-150, and miR-29	Zhou et al., 2016
Human	Macrophages	miR-144	Lv et al., 2016
human	Serum	hsa-let-7b and hsa-miR-30b	Xin et al., 2016
Mouse	Macrophages	let-7e, miR-29a, and miR-886-5p	Sharbati et al., 2011
Human	Macrophages	miR-3179, miR-147, and miR-19b-2^∗^	Yi et al., 2012
Human	Macrophages	miR-155, miR-146a, miR-145, miR-222^∗^, miR-27a, and miR-27b	Furci et al., 2013
Human	Serum	miR-424-5p, miR-493-5p, miR-296-5p, miR-27b-3p, miR-377-5p, miR-3680-5p, and miR-191-5p	Meng et al., 2014
Human	T cells	miR-144^∗^	Liu et al., 2011
Human	Whole blood	miR-424 and miR-365	Wang et al., 2011


In a very recent study, co-regulatory networks consisting of transcription factors and miRNAs as well as their target genes were analyzed from whole blood of TB patients. This TF-gene network showed that *SPI1*, *CEBPB*, *STAT1*, *STAT2*, *STAT3*, *STAT4*, and *STAT5A* directly regulate 22, 12, 11, 1, 10, 1, and 3 genes, respectively. The results suggested that TF-miRNA gene co-regulatory networks may help provide a way to discover future biomarker and therapeutic targets (Lin et al., 2017). Wu et al. (2014) uncovered several miRNA-gene interactions differentiating among active TB, LTBI and healthy subjects. Wang et al. (2015) discovered that expression of miRNA-31 in pediatric TB patients was significantly lower compared with that in normal children. Furthermore, miRNA-31 expression was negatively correlated with serum levels of IL-6, TNF-α, NF-κ0, and IFN-overed several m- analysis study reported that the current data do not support any association between miR-146a/499 polymorphisms and genetic susceptibility of humans to TB (Lu et al., 2016). Latorre et al. (2015) reported nine differentially expressed miRNAs in active TB patients with respect to those with LTBI and healthy controls. Qi et al. (2012) suggested that altered levels of serum miRNAs have great potential to serve as biomarkers for early detection of pulmonary TB. Similarly, Zhou et al. (2016) identified the expression profile of circulating miRNAs and demonstrated that miRNAs may act as effective biomarkers for the early diagnosis of childhood TB.

Barry et al. (2015) examined the appropriateness of 12 miRNAs and RNU6B to normalize circulating plasma miRNA levels in individuals with active TB. These data identify miR-93 as a suitable miRNA that can be used for normalizing miRNA levels in TB patients. Ren et al. (2015) acquired miRNA expression data from sensitive *Mtb* and MDR *Mtb* strains by using next generation sequencing (NGS) and revealed that 142 miRNAs were differentially expressed in the MDR *Mtb* strains but not in sensitive TB strains. This suggests that miRNAs may have a role in the development of drug-resistance in *Mtb* strains. Miotto et al. (2013) identified 15 serum miRNAs as signature in pulmonary TB with a diagnostic accuracy of 82%. These studies have contributed substantially to present the differentially expressed miRNAs as potential biomarker candidates for diagnosis of TB, but there is no established miRNA biomarker so far. Several factors may contribute to this slow discovery, including heterogeneous study designs having small study group sizes, noticeable inter-individual variability of miRNA expression and inadequate statistical evaluation for candidate selection (Ueberberg et al., 2014). Moreover, many other pathological conditions may also induce similar miRNAs expression profiles. Keeping in view these limitations, additional investigations are a prerequisite for the selection and use of miRNAs as biomarkers for diagnosis of TB.

## miRNAs as Regulators of TB Immunity

### Regulation of Inflammatory and Immuno-Modulatory Cytokines

Several miRNAs have been demonstrated to regulate the inflammatory and immune response signaling pathways (**Table 2**) in response to challenge with *Mtb* (Guo et al., 2010; Chatterjee et al., 2011; Fu et al., 2011; Huang et al., 2011; Li et al., 2011; Liu et al., 2011). Innate immune cell activation is regulated by miR-155, miR-146a, miR-21, and miR-9 (Belver et al., 2011). Similarly, miR-155 is a positive regulator of TLR signaling, and is induced upon stimulation of murine macrophages with interferon beta (IFN-β) or TLR ligands (Gantier, 2010; Liston et al., 2010). TNF biosynthesis is inhibited by miR-125b in *Mtb-*infected human alveolar macrophages (Rajaram et al., 2011). miR-29 helps control innate and adaptive immune responses against *Mtb* by targeting interferon-γ and it is suggested as a biomarker for pulmonary tuberculosis because it is related to the clinical manifestation of the disease (Ma et al., 2011).

**Table 2:** miRNAs regulation of host immune response in tuberculosis.

Functions	miRNA	Target	Species examined	Tissue/Cell examined	Reference
Apoptosis	miR-145	TRAF6	Mouse	Stem cells	Starczynowski et al., 2010
	let-7e	Caspase 3	Human	Monocyte derived macrophages	Sharbati et al., 2011
	miR-29a	Caspase 7	Human	Monocyte derived macrophages	Sharbati et al., 2011
	miR-155	FOXO3	Human	Monocytes	Huang et al., 2015
	miR-20a-5p	JNK-2	Human	Macrophages	Zhang et al., 2016
	miR-21	Bcl-2	Mouse	RAW264.7 macrophages	Wang Q. et al., 2014
Cytokines	miR-144^∗^	INF-γ and TNF-α	Human	Whole blood	Liu et al., 2011
	miR-146a	IRAK-1/TRAF-6 pathway	Mouse	RAW264.7 macrophages	Li et al., 2013
	miR-146a	TNF-α	Human	Alveolar macrophages	Liu et al., 2014
	miR-223	CXCL2, CCL3 and IL-6	Mouse	miR-223^-^/^-^ mouse	Dorhoi et al., 2013
	let-7f	A20, TNF, IL-1β	Mouse	RAW264.7 and BMDMs	Kumar et al., 2015
	miR-125b	TNF	Human	Monocyte derived macrophages	Rajaram et al., 2011
	miR-27a	IRAK4	Human	THP-1	Wang et al., 2017
	miR-27a	IL-10 and TAB2	Mouse	RAW264.7 and BMDMs	Hussain et al., 2018
	miR-27a and miR-27b	IRF4	Computational study	TargetScan database	Ahluwalia et al., 2017
	miR-302c	IRF5	Computational study	TargetScan database	Ahluwalia et al., 2017
	miR-155, miR-132, and miR-455-5p	SOCS transcription factors	Computational study	TargetScan database	Ahluwalia et al., 2017
Nitric oxide suppression	miR-146a	NF-kB, MAPK	Mouse	RAW264.7 macrophages	Li et al., 2016
	miR-155	C/EBPb	Mouse	RAW264.7 macrophages	Qin et al., 2016
Increased bacterial survival	miR-155	SHIP1/protein kinase B (Akt) pathway	Mouse	Macrophages	Rothchild et al., 2016
Inhibition of antimicrobial peptides	hsa-miR-21	CYP27B1, IL1B	Human	Monocytes	Liu et al., 2012
Autophagy	miR-155	Rheb	Mouse	RAW264.7 and BMDMs	Wang et al., 2013
	miR-142-3p	N-Wasp	Mouse and human	J774A.1 and primary human macrophages	Bettencourt et al., 2013
	miR-33	ATG5, LAMP1	Human	THP-1 and HEK293 cells	Ouimet et al., 2016
	miR-125a-3p	UVRAG	Mouse	RAW264.7 and J774A.1 macrophages	Kim et al., 2015
	miR-17-5p	ULK-1	Mouse	RAW264.7 macrophages	Duan et al., 2015
	miR-144-3p	ATG4a	Mouse	RAW264.7 macrophages	Guo et al., 2017
	miR-20a	ATG7andATG16L1	Mouse	RAW264.7 macrophages	Guo et al., 2016
	miR-23a-5p	TLR2/MyD88/NF-κB	Mouse	RAW264.7 and BMDMs	Gu et al., 2017
	miR-26a	KLF 4	Mouse	RAW264.7 macrophages	Sahu et al., 2017
	miR-17-5p	Mcl-1/STAT3	Mouse	RAW264.7 macrophages	Kumar et al., 2016


#### Role of miR-146a and miR-155 in Host Immune Response Regulation

With respect to TB, miR-146a and miR-155 are the most vastly studied miRNAs influencing the host–pathogen interaction. miR-146a expression is driven by the transcription factor NF-κB, predicted to base-pair with sequences in the 3′-UTRs of the TNF receptor-associated factor 6 and IL-1 receptor-associated kinase 1 genes (Taganov et al., 2006). Mice deficient in miR-146a showed fatal IFN-γ-dependent autoimmune disease, possibly involving the STAT1 signaling pathway (Lu et al., 2016). miR-146a also represses mycobacteria-mediated inflammatory response and facilitates bacterial proliferation in RAW264.7 macrophages via the IRAK-1/TRAF-6 pathway (Li et al., 2013). In addition, it is overexpressed in macrophages in response to *M. bovis* BCG while downregulated in alveolar macrophages of pulmonary TB patients and negatively regulating TNF-α (Liu et al., 2014). More recently, it has been described that miR-146a promotes mycobacterial survival in RAW264.7 macrophages through suppression of nitric oxide (NO) production (Li et al., 2016).

miR-155 also has been extensively studied and plays an important role in various physiological and pathological processes (Hacker and Karin, 2006; Faraoni et al., 2009; Kutty et al., 2010; Wang et al., 2010; Savan, 2014; Forster et al., 2015). It is induced upon infection of murine macrophages with mycobacteria and known to be a positive regulator of TLR signaling (Wang J. et al., 2014). Pharmacological inhibition of the Janus kinase (JNK) pathway blocked the induction of miR-155 in response to either polyriboinosinic: polyribocytidylic acid or TNF-α, suggesting that miR-155 signals through the JNK pathway (O’Connell et al., 2007). Iwai et al. (2015) unveiled that miRNA-155 knockout mice were susceptible to *M. tuberculosis* infection and died significantly earlier with significantly higher numbers of CFU in lungs, as compared to wild-type mice. These studies demonstrate the protective role of miRNA-155 against mycobacterial infection. On the other hand, miR-155 augments the survival of macrophages thereby providing a niche for the replication of Mtb, it promotes the survival of Mtb-specific T cells enabling an effective adaptive immune response. These effects of miR-155 on innate and adaptive immunity are associated with the observations that miR-155-deficient mice better control early Mtb infection, but are compromised in their ability to control late infection (Rothchild et al., 2016). Furthermore, miR-155 reportedly inhibits apoptosis in monocytes (Huang et al., 2015). Zhang et al. (2015) discovered that there was an inverse relationship between serum miR-155 abundance and NK cell cytotoxicity. Serum miR-155 levels were shown to be negatively associated with the TB-suppressing activity of NK cells. Similarly, Qin et al. (2016) reported that miR-155 expression is significantly increased in macrophages after *M. marinum* infection, resulting in decreased NO synthesis and increased mycobacterium load. These findings support the proposed theory that a single miRNA can target multiple genes at the same time and emphasizing the regulatory role of miRNAs (Mayr et al., 2007; Pillai et al., 2007).

#### Role of Other miRNAs in Host Immune Response Regulation

Besides these two extensively studied miRNAs, many other miRNAs have been reported to play a role in *Mtb* infection. miR-223 has a critical role in the control of TB and potentially other chronic inflammatory diseases. Chemoattractants like CXCL2, CCL3, and IL-6 have been identified as targets of miR-223 in myeloid cells (Dorhoi et al., 2013). Similarly, a transgenic mouse with endogenous blockage of miR-29 showed increased resistance against *Mtb* infection (Ma et al., 2011). A recent study has identified miR-27a as a restrainer of immune response in *Mtb* infection by targeting IRAK4. Expression levels of IFN-γ, IL-β, IL-6, and TNF-α were significantly decreased following transfection of miR-27a mimics (Wang et al., 2017). miRNAs expression and transcriptional regulation of target genes potentially affect the regulation of multiple immunological responses. These studies enhance our understanding of the role of miRNAs in host-pathogen interactions and offer a new way to improve our diagnostic tools and treatments regimes against TB.

### miRNAs as Regulators of Autophagy

Autophagy is an intracellular process involving self-digestion or self-eating, whereby cytoplasmic constituents are transported to and degraded by lysosomes (Lamb et al., 2013). Besides being critical for other cell functions, autophagy plays a key role in immune responses against invading viral and bacterial pathogens (Huang and Brumell, 2014). Similarly, apoptosis is also one of the important host defense mechanisms through which the host cells limit the extent of damage caused by the infection. However, the capacity of *Mtb* to survive and replicate in host macrophages is central to *Mtb* pathogenesis and is often associated with its degree of virulence (Weiss and Schaible, 2015). It is important to explore how *Mtb* hijacks host immunomodulatory signaling pathways to survive and replicate in macrophages for appropriate control and treatment of the disease. It is well established that *Mtb* has developed several schemes to avoid the antimicrobial effects of macrophages to be able to survive intracellularly (Ahmad, 2011). One of these significant strategies is its ability to block phagosome maturation and deploy other countermeasures to impede autophagy, and evade the hostile environment of phagolysosomes (Vergne et al., 2004; Russell, 2011; Espert et al., 2015). Moreover, phagosome maturation and mycobacterial killing can be reinstated through exogenous induction of autophagy in infected macrophages (Deretic et al., 2013).

The pathways associated with autophagy are tightly regulated at the post-transcriptional level and have been well described, but the contribution of miRNAs in activation or inhibition of autophagy during *Mtb* infection had been largely unknown. However, in recent years many research groups have successfully unveiled the role of several miRNAs in autophagy regulation during *Mtb* infection. Ouimet et al. (2016) reported that miR-33 induction in THP-1 and HEK293 cells inhibits the integrated pathways involved in autophagy and also reprograms the host lipid metabolism for intracellular survival and persistence of *Mtb*. Guo et al. (2017) revealed that *M. bovis* BCG infection of macrophages leads to increased expression of miR-144-3p, which induces autophagy-related gene 4a (ATG4a) to inhibit autophagy. In another study, overexpression of miR-23a-5p dramatically prevented *Mtb*-induced activation of autophagy in macrophages by modulating TLR2/MyD88/NF-κB signaling (Gu et al., 2017). Similarly, miRNA-20a targets ATG7 and ATG16L1 and is able to inhibit autophagy (Guo et al., 2016).

In contrast to the inhibitory role of miRNAs in autophagy induction reported by the above studies, other studies have identified several miRNAs involved in enhancing autophagy during *Mtb* infection. Sahu et al. (2017) reported that miR-26a mimic attenuates *Mtb* survival in macrophages by targeting the transcription factor KLF4. This transcription factor is able to prevent trafficking of *Mtb* to lysosomes. miR-155 expression accelerates the autophagy-mediated anti-mycobacterial response by targeting Ras homolog enriched in brain (Rheb), a negative regulator of autophagy (Wang et al., 2013). Similarly, Kumar et al. (2016) demonstrated that a miR-17/PKCδ/STAT3 axis is involved in regulating autophagy during *Mtb* infection. Neural Wiskott-Aldrich syndrome protein (N-Wasp) is an actin-binding protein involved in phagocytosis during microbial challenge. *Mtb* induced expression of miR-142-3p targets N-Wasp leading to reduced phagocytosis (Bettencourt et al., 2013).

### miRNAs as Regulators of Apoptosis

**FIGURE 1:**
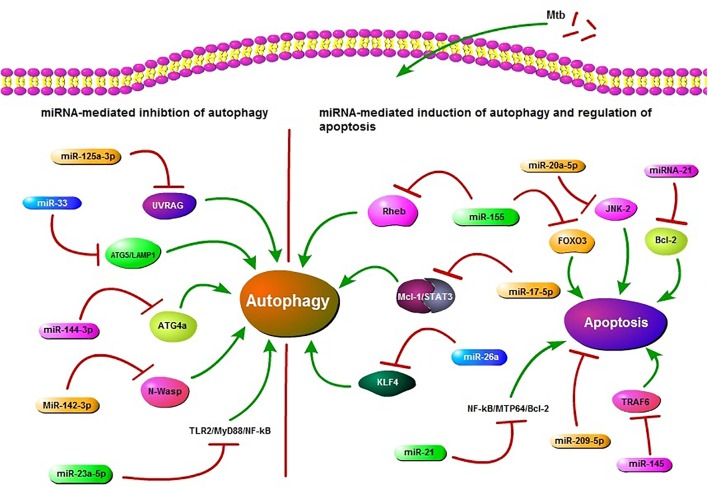
Schematic presentation of miRNA regulation of autophagy and apoptosis. miR-125a-3p, miR-33, miR-144-3p, miR-23a-5p, and miR-142-3p are potential inhibitors of autophagy in *Mycobacterium tuberculosis (Mtb)* infection. miR-125a-3p inhibits autophagy through targeting UV radiation resistance-associated gene (UVRAG), miR-33 targets ATG5/LAMP1, miR-144-3p targets autophagy-related gene 4a (ATG4a), miR-23a-5p inhibits the TLR2/MyD88/NF-κB leading to reduced autophagy and miR-33 also plays an inhibitory role via targeting some unknown factors. miR-142-3p targets Neural Wiskott-Aldrich syndrome protein (N-Wasp) leading to reduced phagocytosis. While miR-155, miR-17-5p, and miR-26a target Ras homolog enriched in brain (Rheb), Mcl-1/STAT3, KLF4, respectively, and play a positive role in autophagy regulation during Mtb infection. miR-155 also inhibits apoptosis by targeting FOXO3. miR-21 targets NF-kB-MTP64-Bcl-2 to down regulate the apoptosis. miR-20a-5p, miR-21, miR-209-5p, and miR-145 also regulate apoptosis. All of these miRNAs may act as potential targets in HDT.

MiRNAs also play a role in regulation of apoptosis in *Mtb* infection. According to recent findings, miR-20a-5p functions as a negative regulator of mycobacterial-triggered apoptosis and inhibition of miR-20a-5p results in more efficient *Mtb* clearance (Zhang et al., 2016). MPT64 is one of the important secreted proteins by *Mtb* and it could inhibit apoptosis of RAW264.7 cells through the NF-kB-miRNA21-Bcl-2 pathway (Wang Q. et al., 2014). Huang et al. (2015) demonstrated that miR-155 targets FOXO3 and regulates apoptosis. Other studies have also reported the role of miRNAs in apoptosis in *Mtb* infection (Starczynowski et al., 2010; Sharbati et al., 2011). Collectively, these findings highlight the role of miRNAs in regulation of autophagy and apoptosis in TB (**Figure 1**). These findings also offer an opportunity for exogenous manipulation of miRNAs to restrain the multiplication and enhance the clearance of *Mtb* from the host cells.

## miRNAs as Therapeutic Targets

Application of miRNAs as a novel class of drug targets for treatment of various diseases is an emerging area of research. For example, miRNAs have been used as therapeutic targets in hepatitis C virus infection, cancer and cardiovascular diseases (Lanford et al., 2010; Takeshita et al., 2010; Kotsinas et al., 2015). Exogenous manipulation in pathologically imbalanced miRNAs is able to transform the phenotype of cancer cells (Merhautova et al., 2016). Moreover, antitumor activity of miRNAs has been demonstrated and safety of use has been established in hepatic carcinoma (Callegari et al., 2015). The expression of miRNAs can be manipulated for therapeutic purposes either through positive or negative regulation. Novel intervention strategies exploit miRNA-mimics to restore optimal miRNA expression or anti-miRNAs to block abnormally produced miRNAs (Stenvang et al., 2012). For example, miRNAs can be targeted therapeutically to inhibit their maturation (Lee et al., 2006). Similarly, it is possible to increase the activity of downregulated anti-mycobacterial miRNAs by using synthetic oligos and reduce the effects of overexpressed pro-mycobacterial miRNAs through antisense oligonucleotides or anti-miRNA complementary to mature miRNA (Meister et al., 2004; Grimm et al., 2006; Baumann and Winkler, 2014). Another alternative strategy is to replace specific miRNA by gene therapy using viral vectors (Uprichard, 2005).

### *In Vivo* Manipulation of miRNAs Expression

Besides these anti-miRNA techniques, substantial advances have been made concerning delivery of miRNAs into lungs and encouraging results have described modulation of TGF-β1 expression in a mouse model of TB (Rosas-Taraco et al., 2011). *In vivo* delivery of miRNAs has been performed by lentiviral vectors, lipid conjugates, or small exosome-like vesicles. Antigen-specific exosome-like nanovesicles have been transfected with mimics or anti-miRNA to restore miRNA expression in target cells (Bryniarski et al., 2013). In a similar study, primary B cells transfected with a specific anti-miRNA have been shown to deliver the molecule successfully to antigen-activated T cells (Almanza et al., 2013). Zheng D. et al. (2015) successfully silenced *in vivo* miR-195 expression by using anti-miR-195. Similarly, *in vivo* inhibition of miR-328 by intra-tracheal inoculation of anti-328 resulted in 4-fold enhanced non-typeable *Haemophilus influenzae* (NTHi) clearance from the lungs as compared to controls (Tay et al., 2015). Thus, miRNAs can be targeted in the lung to enhance host immunity against microbial infections and offer a potential new anti-mycobacterial approach for the treatment of TB. Further studies are required to define the most appropriate route of miRNA delivery and pharmaco-vigilance problems, providing a valid foundation for future miRNA-based HDTs of TB.

### Nanoparticle-Mediated Delivery of miRNAs in TB

Technological developments at the micro and nanoscale can be used to modulate the immune response (Dacoba et al., 2017). Multiple materials and techniques have been developed to deliver drugs and a broad array of cargo to cells, including nanostructured materials (Vázquez-Hernández et al., 2017), synthetic nanoparticles (Ayer and Harm-Anton, 2017), proactive biomimetic delivery systems (Parodi et al., 2017), functional polymers (Jiang et al., 2017) or cell-penetrating peptides (Tashima, 2016), among others. Several materials have been proposed to help treat immune diseases with nanoparticles, including silica, iron oxide, gold, liposomes, or polylactic-co-glycolic acid (PLGA) (Prosperi et al., 2017). Of particular interest in infectious diseases are techniques that can encapsulate miRNAs and deliver them to the cells that are at the forefront of the immune response, i.e., macrophages or dendritic cells, taking advantage of the innate ability of these cells to internalize foreign bodies. The idea of miRNA delivery fits into the general concept of nanoparticle-based induction of immune responses by targeting phagocytes (i.e., macrophages), in contrast to the alternative method of targeting adaptive lymphocytes (i.e., T cells). MiRNA delivery has been explored for many applications related to inflammation (Zhou et al., 2013) and cancer (Fernandez-Piñeiro et al., 2017), and its potential use demonstrated in infectious diseases (Pegtel et al., 2010). MiRNAs can be incorporated into both natural –i.e., chitosan- and synthetic – i.e., (PLGA)- particle-forming materials. For example, nanoparticle- and liposome-loaded miRNAs have been delivered to macrophages (Duan et al., 2015; Moore et al., 2016). At the time of writing, however, very few reports are available on the implementation of this technique in TB. This implementation can be discussed by either describing current general uses of nanoparticles in immunology or broadly mentioning the utilization of nanoparticles with miRNAs. We will briefly combine these two views and describe nanoparticle methods used elsewhere that can be adopted in miRNA delivery in TB.

#### Direct Delivery of miRNAs to Modulate Host Immune Response

To help eliminate or substantially reduce mycobacterial activity by targeting macrophages and dendritic cells, several approaches can be implemented. An obvious approach is to directly deliver miRNAs that improve host immune responses against TB, or more generally that facilitate increased expression of anti-inflammatory molecules like IL-10. Among the candidates for this approach we can cite miR-155, miR-146a, miR-145, miR-99b, miR-19b-2^∗^, miR-27a, or miR-27b. Conversely, a second approach is to deliver siRNAs that help decrease expression of pro-inflammatory cytokines such as TNF-α that are induced by miRNAs. Candidates for this approach are those miRNAs that are overexpressed during TB infection, including let-7e, miR-29a, miR-886-5p, miR-147, or miR-3179. In addition to siRNAs, this approach could include the delivery of small molecules (drugs) that can selectively affect immune responses including dexamethasone or other small molecules which modify the host’s ability to regulate miRNA expression.

Direct miRNA delivery could knock-down, overexpress or downregulate appropriate host molecular targets against TB. An early example of this approach of direct delivery is the nanoparticle release of miR-223 in macrophages to facilitate phenotype transitions (Tran et al., 2016). To implement the direct delivery, the critical aspect is the selection of the miRNAs. When innate immune cell activation is desired, direct delivery of miR-155 may be an option, because of its role as a positive regulator of TLR signaling (Wang J. et al., 2014) and its promotion of autophagy and mycobacterial clearance (Wang et al., 2013). Similarly to miR-155, nanoparticle delivery of miR-125b during *Mtb* infection can be useful in inhibiting TNF biosynthesis. Delivery of these miRNAs have been demonstrated previously in pancreatic cancer cells (Su et al., 2016) or in lung cancer (Talekar et al., 2016), but not in infectious diseases. In general, this approach of direct delivery should be implemented for those miRNAs that are downregulated during TB infection, including miR-155, miR-146a, miR-145, miR-222^∗^, miR-27a, or miR-27b (Spizzo et al., 2010; Belver et al., 2011; McGregor and Choi, 2011; Graff et al., 2012). Only a few reports are available describing nanoparticle uses with mi-R155, miR-223, miR-146, or miR-125b, among the many miRNAs involved in TB cited in our manuscript.

#### Use of siRNAs to Downregulate the miRNAs

In the second approach mentioned –releasing siRNAs to downregulate miRNAs expression-, a clear candidate may be miR-146a, because it facilitates bacterial proliferation via the IRAK-1/TRAF-6 pathway, negatively regulates TNF-α and promotes mycobacterial survival in macrophages through suppression of NO production. Interestingly, nanoparticles loaded with miR-146 have been used to inhibit inflammation in keratinocytes (Urgard et al., 2016) and human dental pulp cells (Liu et al., 2016) and to inhibit kidney fibrosis (Morishita et al., 2015). This suggests that both methodologies –direct delivery and secondary molecular induction- can be complementary approaches in nanoparticle-mediated delivery of miRNAs. Delivery of molecular compounds that facilitate upregulation or downregulation of host’s expression of miRNAs is part of this second approach. For this to work, it would be necessary to know what class of molecules (or particular molecule) can directly regulate the host expression of the targeted miRNAs. In summary, there appears to be great potential for the broad implementation of nanoparticle-mediated release of miRNAs important during TB infection. Additional studies will be necessary to clarify the role of these differentially expressed miRNAs in mycobacterial infection and observe differential effects of direct miRNA delivery or indirect regulation to each particular set of cells.

### Recent Studies Illustrating miRNAs as Potential Therapeutic Targets

In recent years, several research groups have pointed out many miRNAs that can be targeted to treat TB. For instance, miR-155 is negatively associated with the TB-suppressing activity of NK cells (Zhang et al., 2015). The same miRNA also leads to autophagy-mediated mycobacterial elimination by targeting Rheb (Wang et al., 2013). Ouimet et al. (2016) revealed that silencing of miR-33 and miR-33^∗^ by genetic or pharmacological means promotes autophagy flux through depression of key autophagy effectors and AMPK-dependent activation of the transcription factors FOXO3 and TFEB. In a similar study, miR-23a-5p modulated the host innate immune response by promoting *Mtb* survival and inhibiting autophagy induction through TLR2/MyD88/NF-κB pathway (Gu et al., 2017). Cytokines are important mediators of inflammatory and immune responses. miR-99b is able to inhibit the secretion of pro-inflammatory cytokines in *Mtb* infection, suggesting a new approach for designing miRNA-based therapies and control of TB (Singh et al., 2013). miR-206 has been suggested to function as an inflammatory regulator leading to the expression of MMP9 by targeting TIMP3 in *Mtb* infection (Fu et al., 2016). Similarly, Lou et al. (2017) demonstrated that miR-20b can alleviate the inflammatory response in TB mice by targeting the NLRP3/caspase-1/IL-1β pathway. Zhang et al. (2017) showed that the induction of miR-32-5p strongly increases the survival rate of *Mtb* by directly targeting Follistatin-like protein 1 (FSTL1) through the TLR-4/miRNA-32-5p/FSTL1 pathway. However, a possible limitation is that most of the miRNAs are not entirely gene-specific. One miRNA may target multiple mRNAs, suggesting that exogenous miRNA administration might exhibit off-target effects. In reality, the exploitation of miRNAs in HDT is still in its infancy stage but it has opened an exciting avenue for the control and treatment of TB.

## Conclusion and Future Prospects

Tuberculosis (TB) is one of the world’s most deadly communicable diseases and *Mtb* is difficult to eradicate, due to its capability to persist within macrophages. Macrophages play a pivotal role in the host immune response against *Mtb*, a response tightly regulated by multiple factors, including miRNAs. The emerging role of miRNAs in regulating both adaptive and innate immune responses against *Mtb* has attracted increasing attention from many research groups in recent years. Many studies have revealed that differential expression of miRNA could reflect disease progression and may distinguish between active and LTBI. These findings provide insights into the potential utility of miRNAs as biomarkers for the diagnosis of TB. However, a possible limitation in the use of miRNAs as biomarkers is that most of the miRNAs are not completely gene-specific. Active participation of miRNAs in modulation of autophagy and apoptosis against *Mtb*, and the emerging role of nanotechnology in medicine opens an exciting avenue for the exploitation of nanoparticles-mediated delivery of miRNAs in host directed therapy (HDT) against TB.

These findings provide valuable information and a firm foundation for the development of HDT based on miRNAs. Moreover, these advancements in miRNA research have reduced our current limitations related to diagnostics, multidrug resistance and HDT for better control and treatment of TB. In this review, we present a comprehensive survey of the published literature on the differential expression and role of miRNAs, as well as recent advances in the development of miRNA-based biomarkers and therapeutics for TB. Further studies are required to elucidate the full potential of miRNAs as novel TB biomarkers, as well as their manipulation as adjunctive treatment for TB.

## Author Contributions

NS collected the data and wrote the manuscript. TH and SS helped for figure and table compilation. AP assisted in writing the manuscript and provided the critical comments. XZ gave the idea behind the manuscript compilation. DZ reviewed the article before final submission. All authors read and approved the manuscript prior to submission.

## Conflict of Interest Statement

The authors declare that the research was conducted in the absence of any commercial or financial relationships that could be construed as a potential conflict of interest.
